# Lessons for the clinical nephrologist: dialysis decisions in early pregnancy for acute kidney injury due to post-infectious glomerulonephritis (PIGN)

**DOI:** 10.1007/s40620-022-01464-0

**Published:** 2022-10-14

**Authors:** Laura De Souza, Ibrahim Ismail, Robert Baade, Murty Mantha

**Affiliations:** Cairns Base Hospital, 165 Esplanade, Cairns North, QLD 4870 Australia

**Keywords:** Post-infectious glomerulonephritis, Pregnancy, Dialysis

## Case presentation

A 16-year-old Australian Indigenous female at 23 weeks of pregnancy (gravida 1, para 0) presented to the emergency department after 3–4 days of fatigue, vomiting and dark-coloured urine. Aside from multiple skin sores present for some months prior, systemic review was unrevealing. She had no previous medical history. Kidney function tests were normal 6 months earlier. There was a maternal history of rheumatic heart disease but none of kidney disease. The patient had received limited antenatal care. She denied smoking or alcohol use. Her only regular medications were multivitamins and iron.

On examination the patient had a BMI of 17 kg/m^2^. Her blood pressure was 121/79 mmHg, heart rate 95 bpm. She was afebrile with oxygen saturations of 99% on room air. Neurological exam revealed brisk patella reflexes and 1–2 beats of clonus in her right foot. Examination showed the abdomen and uterus were soft. The symphysio-fundal height was 21 cm—appropriate for 23 weeks’ gestation. Bedside fetal ultrasound was performed with note of fetal movements and fetal heart rate of 160 bpm. Morphology echography done externally showed estimated fetal weight above the  90th centile. Skin examination revealed multiple crusted lesions.

Blood tests showed haemoglobin of 10.3 g/dL, normal white cell and platelet counts. Haemolytic screen was reassuring (direct Coombs test and haptoglobin). Serum biochemistry showed potassium of 7.0 mmol/L, bicarbonate of 19 mmol/L, creatinine of 402 micromol/L, and urea of 21.6 mmol/L. Serum albumin was 27 g/L. Urinalysis showed haematuria (erythrocytes > 500 10^6^/L RR with normal cell morphology), and proteinuria with urinary protein creatinine ratio (PCR) 170 g/mol creatinine; cellular casts were not identified.

Subsequent results showed low C3 of 0.13 g/L (RR 0.88–2.01 g/L) with normal C4 complement levels. The anti-DNase was 565 units/mL, and ASOT 210 units/mL. The remaining vasculitic and autoimmune screen was negative. Serum creatinine kinase (CK) was 389 (reference 28–142 UI/L) and urine myoglobin was absent. Ultrasound imaging was normal. Her chest radiograph showed bilateral pleural effusions. Skin swabs cultured non-multi-resistant *Staphylococcus aureus* and *Streptoccocus pyogenes.* Echocardiogram was normal.

Initial management was supportive for control of hyperkalemia, volume overload and infection, with intravenous insulin and glucose, diuretics, and bicillin. On day 6 of hospital admission, she developed severe hypertension with suboptimal response to intravenous frusemide and oral antihypertensives (labetalol and nifedipine)—leading to initiate emergent haemodialysis via non-tunnelled vascular catheter. She received 8 sessions of haemodialysis over a period of 2 weeks.

## Lessons for the clinical nephrologist

Post-infectious glomerulonephritis (PIGN) is an immune-mediated disease due to the deposition of immune complexes and autoimmune reactivity, and commonly associated with skin infections, most commonly due to *Staphylococcus aureus* and *Streptococcus pyogenes*. The spectrum of disease ranges from acute nephritic and or nephrotic syndromes to rapidly progressive kidney failure requiring dialysis. Life-threatening complications related to accelerated hypertension, such as hypertensive encephalopathy and acute pulmonary oedema are a matter of concern.

Importantly, acute PIGN occurs in childhood and in adults, and can predispose to chronic kidney disease [1].

Incidence of PIGN among Australian communities, specifically Indigenous people, is amongst the highest in the world [2, 3]. Recent clusters of PIGN over the last 12 months were described from Kowanyama, a remote community in the northernmost part of the Australian state of Queensland [4]. Our patient had recently relocated from remote Far North Queensland. Young females of childbearing age represent a significant proportion of this at-risk group, and hence management of pregnancy and PIGN assumes clinical significance with respect to maternal, fetal outcomes and decisions surrounding kidney biopsy and dialysis.

PIGN in pregnancy is rare, with a small number of case reports published as early as 1980 by Singson et al. [5]. The rarity of cases in pregnancy is possibly due to the young age group seen in the majority of cases of PIGN, but also begs the question as to whether autoimmune mechanisms specific to PIGN are dampened—as are other autoimmune diseases [6]. Gestation of the reported cases ranged from 17 to 37 weeks at diagnosis with one case occurring in the post-partum period.

Kidney biopsy can be safely performed in the first or early second trimester but is only recommended if the result is likely to alter the management [7, 8]. In this particular case it was not considered necessary. Clinical history and investigations excluded the most important non-glomerular (acute tubular necrosis, acute interstitial nephritis) and glomerular (ANCA-associated vasculitis, anti-GBM disease) causes of kidney injury. In the context of the epidemiological setting and positive skin microbiology for *Streptococcus*, the likely diagnosis of post-infectious glomerulonephritis was supported. Coincident, active infection with *Staphylococcus aureus* likely also contributed to an immune complex-mediated pathology.

There is a paucity of data to guide haemodialysis treatment in pregnancy with acute kidney injury. Targets for blood urea nitrogen (BUN) have been described for pregnancy management in general, with cohort studies suggesting improved fetal outcomes—particularly birth weight and gestational age—when mid-week maternal BUN is less than 35–48 mg/dL (approximately equating to serum urea 12 mmol/L) [9]. The timeline in this case—particularly the several days’ delay from admission to dialysis start—is a key point to highlight. The decision to start dialysis in our case was based on the presence of life-threatening fluid overload, and was not influenced by urea control for fetal protection. This key point may be a matter of further discussion.

Our patient remained hospitalized 2 weeks following last dialysis session, and was discharged with a serum creatinine of 80 micromol/L and urea 5.2 mmol/L. Follow up in the Renal Outpatient Clinic 3 weeks later showed blood pressure of 118/88 mmHg on 20 mg of nifedipine once daily; she was euvolemic, all skin lesions had healed and kidney function remained normal. She subsequently returned to deliver a healthy 2.6 kg female via spontaneous vaginal birth at 37 + 5 weeks. In the months post-partum she maintained normal kidney function, complement levels returned to the normal range and urine protein excretion normalized (Table 1).

**Table 1:** Timeline of investigation results

	Day 0	Day 7	Day 23	3 months later	4 months later	1 year later
	At presentation	When dialysis commenced	At discharge	At follow up	At delivery	
Serum creatinine (umol/L)	402	219	80	48	51	66
Serum urea (mmol/L)	21.6	14.9	5.2	4.4	3.5	7.2
Serum albumin (g/L)	27	20	24	24	22	36
Urine protein-creatinine ratio (uPCR) (g/mol creat)	170	679	260	240	N/A	8

- In summary, PIGN should be considered early as a differential diagnosis, in particular for patients from remote communities in Australia where the incidence of this disease is considerably higher than in other parts of the world.
- Dialysis should be considered for all pregnant women with severe acute kidney injury irrespective of underlying diagnosis, when severe symptoms occur, like in this case. The issue whether serum urea reduction may be a reliable marker to improve fetal outcomes needs dedicated studies.

## Supplementary Information

Below is the link to the electronic supplementary material.

Supplementary file1 (DOCX 14 kb)

## References

[1] Australia’s health 2020 data insights. Australian Institute of Health and Welfare.; 2020. Contract No.: Australia’s health series no. 17. Cat. no. AUS 231. Canberra: AIHW.

[2] Dowler J, Wilson A (2020). Acute post-streptococcal glomerulonephritis in Central Australia. Aust J Rural Health.

[3] Davidson L, Bowen AC (2020). Skin infections in Australian aboriginal children: a narrative review. Med J Aust.

[4] Health authorities managing infectious skin condition at Kowanyama. 2020. https://www.health.qld.gov.au/torres-cape/html/news/health-authorities-managing-infectious-skin-condition-at-kowanyama.

[5] Singson E, Fisher KF, Lindheimer MD (1980). Acute poststreptococcal glomerulonephritis in pregnancy: case report with an 18-year follow-up. Am J Obstet Gynecol.

[6] Adams Waldorf KM, Nelson JL (2008). Autoimmune disease during pregnancy and the microchimerism legacy of pregnancy. Immunol Investig.

[7] Wiles K, Chappell L, Clark K, Elman L, Hall M, Lightstone L (2019). Clinical practice guideline on pregnancy and renal disease. BMC Nephrol.

[8] Hladunewich MA, Bramham K, Jim B, Maynard S (2017). Managing glomerular disease in pregnancy. Nephrol Dial Transplant.

[9] Luders C, Titan SM, Kahhale S, Francisco RP, Zugaib M (2018). Risk factors for adverse fetal outcome in hemodialysis pregnant women. Kidney Int Rep.

