# Supplementary material for: Lessons for the clinical nephrologist: dialysis decisions in early pregnancy for acute kidney injury due to post-infectious glomerulonephritis (PIGN)
Source: J Nephrol. 2022 Oct 14;35(9):2399–401. doi: 10.1007/s40620-022-01464-0 (PMC9700564; doi:10.1007/s40620-022-01464-0)
Supplement: Supplementary file 1 — Supplementary file1 (DOCX 14 kb) [file 40620_2022_1464_MOESM1_ESM.docx]

Supplementary references

1. HDJ DZ, RM W, T P. Post Infectious Glomerulonephritis–Rare, but Reversible Cause of Acute Kidney Injury During Pregnancy. J Immuno Biol; 2017.

2. Hamouda M, Skhiri H, Toumi S, Aloui S, Ahmed L, Ben Dhia N, et al. Post-infectious glomerulonephritis: unusual etiology of postpartum acute renal failure. Nephrol Ther. 2013;9(4):228-30.

3. Nayak SG, Satish R, Kedley P, Deshpande R, Gokulnath, Garg I. Acute renal failure during pregnancy--an unusual cause. Nephrol Dial Transplant. 2007;22(1):264-5.

4. Fervenza F, Green A, Lafayette RA. Acute renal failure due to postinfectious glomerulonephritis during pregnancy. Am J Kidney Dis. 1997 Feb;29(2):273-6. doi: 10.1016/s0272-6386(97)90040-8. PMID: 9016900.
